# Morphology and Anatomy of Branch–Branch Junctions in *Opuntia ficus-indica* and *Cylindropuntia bigelovii*: A Comparative Study Supported by Mechanical Tissue Quantification

**DOI:** 10.3390/plants10112313

**Published:** 2021-10-27

**Authors:** Max D. Mylo, Linnea Hesse, Tom Masselter, Jochen Leupold, Kathrin Drozella, Thomas Speck, Olga Speck

**Affiliations:** 1 Plant Biomechanics Group @ Botanic Garden, Faculty of Biology, University of Freiburg, Schänzlestraße 1, D-79104 Freiburg, Germany; linnea.hesse@biologie.uni-freiburg.de (L.H.); tom.masselter@biologie.uni-freiburg.de (T.M.); thomas.speck@biologie.uni-freiburg.de (T.S.); olga.speck@biologie.uni-freiburg.de (O.S.); 2 Cluster of Excellence *liv*MatS @ FIT—Freiburg Center for Interactive Materials and Bioinspired Technologies, University of Freiburg, Georges-Köhler-Allee 105, D-79110 Freiburg, Germany; 3 Department of Diagnostic and Interventional Radiology, Medical Physics, Medical Center—University of Freiburg, Faculty of Medicine, University of Freiburg, Killianstraße 5a, D-79106 Freiburg, Germany; jochen.leupold@uniklinik-freiburg.de; 4 Faculty of Environment and Natural Resources, Bertoldstraße 17, D-79098 Freiburg, Germany; kathrin.drozella@fobot.uni-freiburg.de; 5 Freiburg Materials Research Center (FMF), University of Freiburg, Stefan-Meier-Straße 21, D-79104 Freiburg, Germany

**Keywords:** Opuntioideae, abscission, cacti, magnetic resonance imaging, periderm formation, tissue tensile testing

## Abstract

The Opuntioideae include iconic cacti whose lateral branch–branch junctions are intriguing objects from a mechanical viewpoint. We have compared *Opuntia ficus-indica*, which has stable branch connections, with *Cylindropuntia bigelovii*, whose side branches abscise under slight mechanical stress. To determine the underlying structures and mechanical characteristics of these stable versus shedding cacti junctions, we conducted magnetic resonance imaging, morphometric and anatomical analyses of the branches and tensile tests of individual tissues. The comparison revealed differences in geometry, shape and material properties as follows: (i) a more pronounced tapering of the cross-sectional area towards the junctions supports the abscission of young branches of *C. bigelovii*. (ii) Older branches of *O. ficus-indica* form, initially around the branch–branch junctions, collar-shaped periderm tissue. This secondary coverage mechanically stiffens the dermal tissue, giving a threefold increase in strength and a tenfold increase in the elastic modulus compared with the epidermis. (iii) An approximately 200-fold higher elastic modulus of the vascular bundles of *O. ficus-indica* is a prerequisite for the stable junction of its young branches. Our results provide, for both biological and engineered materials systems, important insights into the geometric characteristics and mechanical properties of branching joints that are either stable or easily detachable.

## 1. Introduction

The plant family of the Cactaceae with its succulent growth forms and adaptations to habitats with little or only seasonally available water have fascinated researchers for centuries. Cacti differ greatly in their outer appearance; species can be found with unbranched to strongly branched columnar, tree-like, creeping, epiphytic and climbing habits [1]. However, they are united by xerophytic adaptations such as succulence, the absence or the early abscission of leaves (with the exception of the Pereskioideae) and extensive but shallow root systems [1,2]. Within the Cactaceae, the Opuntioideae represent the second largest subfamily with over 300 species [3].

A remarkable feature of the Opuntioideae is the outer geometry of their jointed branches, which is a characteristic and name-giving feature of their tribes. Opuntieae, also referred to as Platyopuntias, grow flat branches called cladodes, whereas Cylindropuntieae grow almost cylindrical, radially symmetric branches called joints [4]. For the sake of simplicity, we will refer to these structures as branches in both tribes. Anatomically, most species of the Opuntioideae are characterised by a densely packed, multiseriate hypodermis, which has crystalline inclusions and which is about twice as thick in species of the Opuntieae as it is in those of the Cylindropuntieae [5]. In addition, a thickened palisade cortex is typical, which, however, is not as distinct as in the related subfamily Cactoideae [6]. Mucilage cells or canals with intercellular secretion are often present, the latter especially in species with flat branches [6,7,8]. The structure of the water-conducting tissue of Opuntioideae varies markedly amongst its species. In addition to vessels and fibres, water-retaining and more flexible wide-band tracheids can frequently be found in the vascular bundles [4,9,10]. In some species a dimorphism occurs in which wide-band tracheids are initially developed, and then the cambium changes its derivatives and develops vessels, parenchyma and/or fibres in later stages [9].

Further differences within the Opuntioideae are found regarding their type of propagation: The main reproductive mode of most species is sexual via seeds, e.g., in *Opuntia ficus-indica* (hereafter: *O. ficus-indica*), which is known for its edible prickly pear fruits [11]. However, some species predominantly propagate vegetatively [12,13] or combine sexual and asexual reproduction [12,14,15]. Asexual propagation is mainly found in species of the Cylindropuntieae. The lateral branches of *Cylindropuntia bigelovii* (hereafter: *C. bigelovii*), for example, fall off easily or stick to the skin or fur of passing vertebrates (by means of their retrorse spines), are carried away, grow roots and mature into new, genetically identical individuals [2]. Nevertheless, this reproduction mode can also be found among the genus *Opuntia* [11,16]. An enzymatic influence has not yet been described in this process of branch detachment in cacti. According to Addicott [17], abscission, i.e., the shedding of plant organs from the remainder of the plant body, can also be the result of purely mechanical forces (e.g., their dead weight or passing animals), as in the illustrated example of *C. bigelovii*.

Regardless of their variable outer morphology, the internal branch structure among the Opuntioideae is similar and is characterised by a net-like arrangement of vascular bundles [12]. Another common characteristic is that the junctions between two (lateral) branches possess a much smaller cross-sectional area than the branches themselves, making them the mechanical weak points of the plant [18,19]. Bending tests for the mechanical quantification of these branch–branch connections have been carried out for several species: The branches of *O. ficus-indica* experience much less deflection at the same applied load than those of their hybrid *O. occidentalis* [20], which is also recognised for its vegetative propagation through shed branches. Older *Opuntia* junctions generally possess a greater stability than younger ones, as has been revealed by an increasing stiffness gradient from apical/lateral to more basal junctions [21]. Similar bending tests on four species of *Cylindropuntia* have shown that junctions of *C. bigelovii* require the least amount of force per junction unit to lead to failure. At the same time, their junctions are the stiffest (but most brittle) of all tested species. In addition, the rooting ability of shed branches is highest for *C. bigelovii*, which is a characteristic of successful vegetative propagation [19].

Although important contributions to the understanding of the mechanics under bending load and the internal structure and anatomy of these junctions in various columnar cacti have been published [22,23], no three-dimensional visualisation of the tissue distribution in Opuntioideae can be found in the literature. Moreover, information concerning the mechanical properties of the tissues involved in the materials systems of the branch junctions has so far only been available with regard to the dry state [24,25,26,27].

The aim of this study was to find answers to the following question: “What are the most influencing factors leading to the different mechanical behaviours of the branch–branch junctions in the Opuntioideae?” For this purpose, we exemplarily analysed *O. ficus-indica,* which is characterized by its stable junctions and which reproduces primarily sexual via fruits/seeds. In addition, we selected *C. bigelovii,* which is known for the abscission of their lateral branches, frequently leading to vegetative propagation via offshoots. Within this study, we have addressed the following three main aspects: (i) quantitative comparisons of the geometry, size and shape of the junctions and lateral branches; (ii) microscopic imaging and magnetic resonance imaging (MRI) scans of entire branch–branch sites to identify the tissues involved and to visualise their 3D spatial arrangement; and (iii) tensile tests on fresh dermal tissues and vascular bundles to characterise their mechanical properties. The interplay of these results, together with the knowledge gained by previous research, should help to gain a broader understanding of the stability of the branch–branch junction in the Opuntioideae.

## 2. Materials and Methods

### 2.1. Plant Material and Cultivation

**Figure 1:**
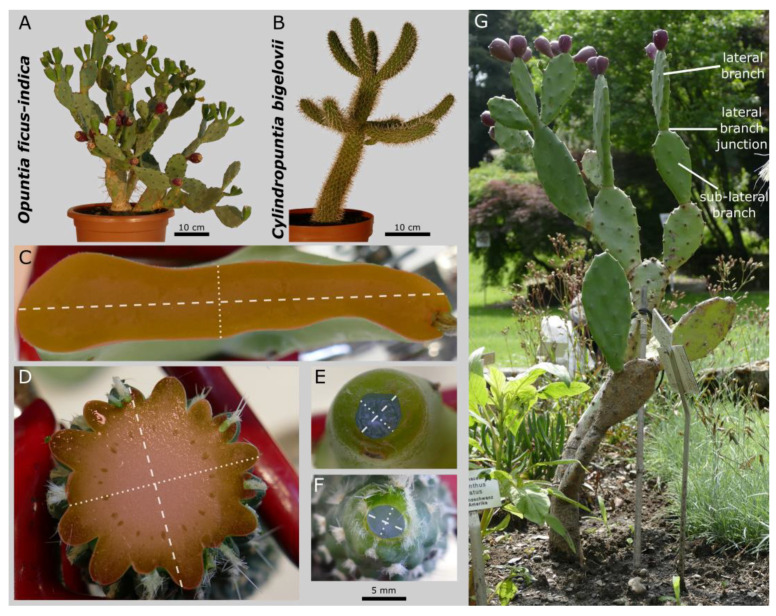
Experimental plants of *Opuntia ficus-indica* (**A**) and *Cylindropuntia bigelovii* (**B**) and their branch and junction cross-sections (*O. ficus-indica*: (**C**,**E**) and *C. bigelovii*: (**D**,**F**)). Exemplary representation of the maximum cross-sectional areas of the branches ((**C**,**D**); coloured red; areoles left out) and the areas of the junction zone ((**E**,**F**); coloured blue). The major axis (dashed white line; defined as the longest axis through the centroid) and the minor axis (dotted white line; orthogonal to the major axis through the centroid) of the respective cross-section are marked. The scale bar applies to subfigures (**C**–**F**). An *Opuntia* plant from the Botanic Garden Freiburg (not used as the experimental plant) used to indicate the lateral and sub-lateral branches and the lateral branch junction (**G**).

Experimental plants of *Opuntia ficus-indica* (L.) Mill. and *Cylindropuntia bigelovii* (Engelm.) F.M. Knuth (Figure 1A,B) were purchased from Kakteenland Steinfeld (Steinfeld, Germany) and assigned with an accession number of the Botanic Garden Freiburg, Germany, where the plants are now cultivated (*O. ficus-indica*: 5400-01; *C. bigelovii*: 5400-02). The plants were cultivated in a phytochamber at the University of Freiburg under controlled conditions (see Methods Section in [28] for more details). All experimental plants were in a well-watered condition at the time of sampling. Plants for the mechanical testing of the tissues were watered one or two days before the experiments. For a comparison of similarly developed plants, only branches that had previously shed their leaves were selected for analyses.

### 2.2. Molecular Genetic Determination

We sequenced the DNA of a representative sample from the tested *Opuntia* plants because of the frequent hybridisation, polyploidisation, morphological variability and accompanying taxonomic difficulties associated with the Opuntieae [29,30] and because the experimental plants had been multiplied by offshoots. Sequencing was not necessary for *C. bigelovii*, as the experimental plants could be definitively identified by conventional methods. Detailed information on the DNA extraction and sequencing can be found in Supplementary Materials Document S1.

### 2.3. Morphometric Analyses

For each species, 12 lateral branches were removed by being twisted off. All samples were taken from branches without peridermal coverage on their surface and were collected from 1 plant of *O. ficus-indica* and from 2 plants with 6 branches each of *C. bigelovii*. The position with the maximum diameter of the separated branch was determined by means of a calliper (Mitutoyo Absolute Digimatic, measuring accuracy: ±0.03 mm, Kawasaki, Japan) and the branch was cut at this point orthogonally to its longitudinal axis by using a razor blade. The resulting branch cross-section and the severed junction at which the branch had been connected were photographed together with a reference scale. The outline of the cross-sections was circled manually (the areoles were left out and, for the junction, only the area that physiologically connected the two branches was included) in ImageJ (version 1.52e; National Institutes of Health, Bethesda, MD, USA), and the cross-sectional area was measured (Figure 1C–F). The ratio of the two areas was calculated using the following Equation (1):

(1) $$Area\;ratio\;\left[\;\right]=\frac{Junction\;area\;\left[mm\right]}{Maximum\;cross-sectional\;area\;\left[mm\right]}$$

The images of the junctions and maximum cross-sections were converted into their binary form in order to measure the major axis (longest axis through the centroid; dashed lines in Figure 1C–F), the minor axis (orthogonal to the major axis through the centroid; dotted lines in Figure 1C–F) and the axial second moments of area with respect to these axes (*I_major-axis_* and *I_minor-axis_* [mm^4^], respectively) by using the BoneJ plugin function “Slice Geometry” in ImageJ [31]. The ratios of the second moments of area were calculated both for the junction and the branch (*I_major-axis_*/*I_minor-axis_* [ ]) and between the junction and branch (e.g., *I_major-axis(junction)_*/*I_major-axis(branch)_* [ ]).

To calculate a variable that indicates the resistance to torsional deformation, we calculated the torsion constant *K* [mm^4^]. For circular cross-sections, this value is equal to the polar second moment of area, although it can also be applied to other cross-section geometries and allows comparisons to be made between them. For the cross-section of the junction of both species and the branch cross-section of *O. ficus-indica*, the torsion constant was obtained by approximating it as an ellipse to enable it to be calculated according to the following Equation (2):

(2) $$K_{ellipse}\;\left[mm^{4}\right]=\frac{\pi \times a^{3}\times b^{3}}{a^{2}+b^{2}}$$

with *a* representing the radius of the major axis and *b* the radius of the minor axis. For the star-shaped cross-section of the branches of *C. bigelovii*, no formula for calculating the torsion constant was available. As an approximation, a circular geometry was assumed, and its equivalent radius *r* was calculated from the measured area, which was inserted into the formula of the torsion constant for circular cross-sections as shown in Equation (3):

(3) $$K_{circle}\;\left[mm^{4}\right]=\frac{\pi \times r^{4}}{2}$$

Figuratively speaking, this assumption flattens the tubercles and applies them to the inner circle, resulting in a slight overestimation of the torsion constant. The ratio for the torsion constant (*K_junction_*/*K_branch_* [ ]) of junction and branch was then calculated.

### 2.4. Magnetic Resonance Imaging

To analyse the 3D arrangement of the tissues, we used the magnetic resonance imaging (MRI) technique, which has been greatly simplified by the technological advances made in recent years. For MRI scans, two samples per species were selected that had one or two lateral junctions and the lateral (outermost) and sub-lateral (second-outermost) branches connected by the lateral junction(s) (Figure 1G). Samples were chosen that lacked periderm formation on the lateral branches. The break-off site of the sub-lateral branch was sealed with Parafilm^®^ (Bemis, Neenah, WI, USA) for transportation. Directly before the scan, the respective sample was cut to the size of the measuring coil (serving as a radiofrequency antenna that receives the MRI signal via electromagnetic induction; with an inner diameter of 7 cm) with a scalpel and the injured sections were sealed with Parafilm^®^. All spines were carefully removed using a nail clipper. MRI was performed on a Bruker Biospec 94/20 small animal scanner, equipped with a Tx/Rx quadrature volume coil (further referred to as “overview scan”), optionally together with a four-element receive-only surface coil (one scan per species; further referred to as “surface coil scan”). The imaging sequence was a 3D gradient echo sequence (FLASH), with resolution up to 67 µm and total acquisition time up to 13 h 33 min (see Supplementary Materials Table S7 for an overview of detailed imaging settings). The recorded data were segmented and visualised using Avizo software (version 2020.2; Thermo Fischer Scientific, Waltham, MA, USA). For segmentation, semi-automated tissue classification was carried out based on the grey values of the image raw data. The resulting tissue geometries were smoothed over 2 or 3 pixels (constrained smoothing algorithm).

### 2.5. Anatomical Analyses

Because of the large amounts of mucilage and the enormous difference in strength between the dermal tissue and the cortex, especially in *C. bigelovii*, different embedding, sectioning and staining techniques were used for the two species. The sections of *O. ficus-indica* were 60–70 µm thick, cut with a cryotome and were stained with safranin (staining of lignified cell walls and nuclei) and astra blue (cellulose). The sections of *C. bigelovii* were 3 µm thick, cut with a rotation microtome and were stained with safranin, acriflavin (DNA), acid yellow (proteins and nuclei) and methylene blue (membranes, protein-rich plasma, tannin-rich cells). The detailed specification of the anatomical methods used can be found in Supplementary Materials Document S2.

Images were recorded using a bright-field microscope (Olympus BX61, Olympus Corporation, Tokyo, Japan) equipped with a microscope camera (DP71, Olympus Corporation, Tokyo, Japan) and CellˆP imaging software (Version 2.6, Olympus Soft Imaging Solutions GmbH, Münster, Germany).

### 2.6. Tissue Mechanics

Mechanical tissue characterisation was carried out by tensile tests. Twelve samples (one sample per branch, divided between two plants, each with six tested branches) were tested with respect to the following tissues: dermal tissue consisting of epidermis and hypodermis (green branches without periderm coverage for *O. ficus-indica* and *C. bigelovii*); dermal tissue fully covered with periderm (only *O. ficus-indica*); vascular bundles of young terminal branches (green branches without periderm coverage for *O. ficus-indica* and *C. bigelovii*) and older, more basal branches (stems almost completely covered with periderm only for *O. ficus-indica*, as none of our experimental plants of *C. bigelovii* had sufficient periderm formation to collect samples for proper testing). For *O. ficus-indica*, a distinction was also made between longitudinal and transversal sampling of the dermal tissues with regard to the main axis of the branch. This was not possible for *C. bigelovii* because of the branch geometry having a large number of tubercles. The dermal tissue samples of *C. bigelovii* were sampled approximately along the longitudinal branch axis. All samples were dissected using a scalpel. Care was taken that no injury to the tissue to be tested was caused, that as little parenchyma tissue as possible was still attached to the samples and that no areolar structures were present in the samples of the dermal tissues.

The minimum cross-section of the tested dermal tissue was approximated as a rectangle and was calculated from the initial width and the thickness of the tested sample. The width was measured at one to three positions, depending on the size of the sample by means of a digital calliper. The thickness of a severed end piece of the sample was measured at two positions by using a stereomicroscope (Olympus SZX9, Olympus Corporation, Tokyo, Japan) equipped with a camera (Color View II, Olympus Soft Imaging Solutions GmbH, Münster, Germany) and CellˆD imaging software (Version 2.6, Olympus Soft Imaging Solutions GmbH). The diameter of the vascular bundle was measured at three positions on a severed end piece by using an Olympus microscope and camera. Assuming a circular geometry, the cross-sectional area was calculated based on this diameter. In cases of multiple measurements for one variable, the lowest value was used for calculation of the cross-sectional area, as failure in the tensile test would most likely occur at this point.

The samples were glued (Loctite 401, Henkel AG & Co. KGaA, Düsseldorf, Germany) on small metal plates immediately after their removal from the branch. For the vascular bundles of *O. ficus-indica*, the adhesive required about 5 to 10 min drying time to prevent slipping effects during testing caused by disintegration of the vascular bundles with the surrounding parenchyma. Samples were kept moist during the drying of the glue by means of a custom-built humidifier, which permanently sprayed atomized water. For all other samples, the glue adhered within a few seconds. The plates were attached to a custom-built tensile device (Technical workshop, Institute of Biology II/III, University of Freiburg, Germany) equipped with a 10 N sensor (50 N for dermal tissue samples with peridermal coverage of *O. ficus-indica*) and the initial length of the sample was measured using the digital calliper. Tensile speed was set to 0.5 mm/s, with displacement and force recorded at 100 Hz. Any preloads were deducted from the data by using a tare force value defined as the last 20 values of the measurement after complete sample failure. Samples that failed on the metal plate or directly adjacent to the gluing site were excluded from further analysis. From the resulting stress (force per cross-sectional area) and strain (elongation relative to initial length) data, we calculate the elastic modulus (the slope of the linear-elastic range in the stress-strain curve), the maximum tensile strength, the strain at failure and the fracture energy (the discrete integral under the force-displacement curve calculated using the trapezoidal rule, relative to the initial cross-sectional area). A camera (Lumix DMC-FZ1000, Panasonic Corporation, Kadoma, Japan) mounted vertically above the tensile test stage recorded videos of the dermal tissue samples during tensile loading. Single images prior to testing and shortly before failure were extracted. The length (*l*) of the sample and width at half length (*w*) were measured using ImageJ software to calculate the respective Poisson’s ratios (*v*) as shown in Equation (4):

(4) $$v=-\frac{\Delta w/w}{\Delta l/l}$$

### 2.7. Statistics

For ease of comparison, all data, regardless of their distribution function, are presented as median values with respective interquartile ranges (IQR; indicated in round brackets). Data processing, data visualisation and statistical analyses were performed with GNU R v.3.6.1 [32], including the packages car [33], readxl and DescTools [34]. Once the assumptions for normally distributed data (Shapiro–Wilk test; α = 0.05) and homoscedasticity (Levene test; α = 0.05) had been checked, datasets from the different plants of one species (for morphometric data of *C. bigelovii* and mechanical data of both species) were tested for significant differences by using the Welch two sample *t*-test (normally distributed data with equal variances) or Wilcoxon rank sum test (not normally distributed data or data with unequal variances). Data of a variable were pooled if no significant differences (α = 0.05) were found between the samples of the two tested plants. Microsoft Excel (version 2016, Microsoft Corporation, Redmond, WA, USA) was used to calculate descriptive statistics. Because of different sample sizes and some non-normally distributed data sets, the Kruskal–Wallis test was used to test for differences for all comparisons between the different mechanical data groups of dermal tissues and vascular bundles (α = 0.05). As a post-hoc test, we used the pairwise Wilcoxon rank-sum test with the Holm correction. The Welch two sample t-test was employed to test for differences between the mechanical data of the two species (as all data sets were normally distributed and equality of variances was given). Levels of significance were as follows: *p* ≥ 0.05: not significant (n.s.); 0.05 > *p* ≥ 0.01: significant (*); 0.01 > *p* ≥ 0.001: very significant (**); 0.001 ≥ *p*: highly significant (***).

## 3. Results

### 3.1. Molecular Genetic Determination

No irregularities occurred during the DNA extraction and sequencing of the *Opuntia* sample. The chromatogram of the issued sequence showed well-resolved, distinct peaks with no background noise; all bases could be clearly determined (see Supplementary Materials Document S1.2 for detailed information). The classification as *Opuntia ficus-indica* could be confirmed with the help of a database comparison. A complete match with entries of South Korean samples (direct submissions by In & Lee 2006 (entry accession no. AB250211.1) and Yang 2020 (accession no. MW036310.1, MW036309.1, MW036308.1 and MW036304.1) was detected, which implied that the examined plant samples originated from the clonal reproduction of plants cultivated in South Korea.

As morphological similarity was noted to the closely related species *Opuntia tomentosa*, *Opuntia megacantha* and *Opuntia schumanii*, the sequence of the presumed *Opuntia ficus-indica* was aligned with the database entries of those species (see Supplementary Materials Document S1.3 for detailed information). Based on at least five sites of complete discrepancy (positions 108, 370, 430, 539, 581), the sample was distinguished from all given reference sequences.

### 3.2. Morphometric Analyses

In order to quantify the taper represented by the decrease of the cross-sectional area of the maximum cross-sectional area of lateral branches to their junctions, the respective areas were determined morphometrically, and their ratio was calculated. In addition, the axial second moments of area (along the major and minor axes) and the torsion constant for both cross-sections were determined. No significant differences were found between the maximum branch cross-sectional areas, with a median value of about 3.5% larger value for *C. bigelovii* than for *O. ficus-indica*. Significantly higher values for *O. ficus-indica* were found for the junction area between the lateral and the sub-lateral branches (by about 62.3%) and the area ratio (by about 78.9%), establishing a significantly smaller taper in the latter species (Table 1).

For the junction, the median values of the axial second moments of area along the major axis (by a factor of 2.2) and the minor axis (by a factor of about 2.1) were significantly larger for *O. ficus-indica* than for *C. bigelovii*. However, their ratios did not differ significantly between the species. For the maximum branch cross-sections, the median values of the second moments of area for *C. bigelovii* were similar (with an *I_major-axis_*/*I_minor-axis_* ratio of about 0.94), whereas those for *O. ficus-indica* differed greatly (with an *I_major-axis_*/*I_minor-axis_* ratio of about 0.02). The median values of *I_minor-axis_* were significantly larger for *O. ficus-indica* (by a factor of about 7.4), whereas those of *I_major-axis_* were significantly larger for *C. bigelovii* (by a factor of about 6.2).

The median values of the torsion constant of the junctions were significantly larger for *O. ficus-indica* (by a factor of about 2.6), whereas those of the maximum branch cross-section were significantly larger for *C. bigelovii* (by a factor of about 5.2). For both species, the values of *I_major-axis_*, *I_minor-axis_* and the torsion constant *K* of the branch, at its maximum width, were at least a factor of 46 higher than those of the junction. Particularly large differences were found for *I_minor-axis_* of *O. ficus-indica* (by a factor of about 1736) and for *I_minor-axis_* (by a factor of about 502), *I_major-axis_* (by a factor of about 644) and the torsional constant *K* (by a factor of about 576) of *C. bigelovii* (Table 1). The raw data and detailed statistical analyses for all morphological variables can be found in Supplementary Materials Table S8.

### 3.3. MRI Analyses

**Figure 2:**
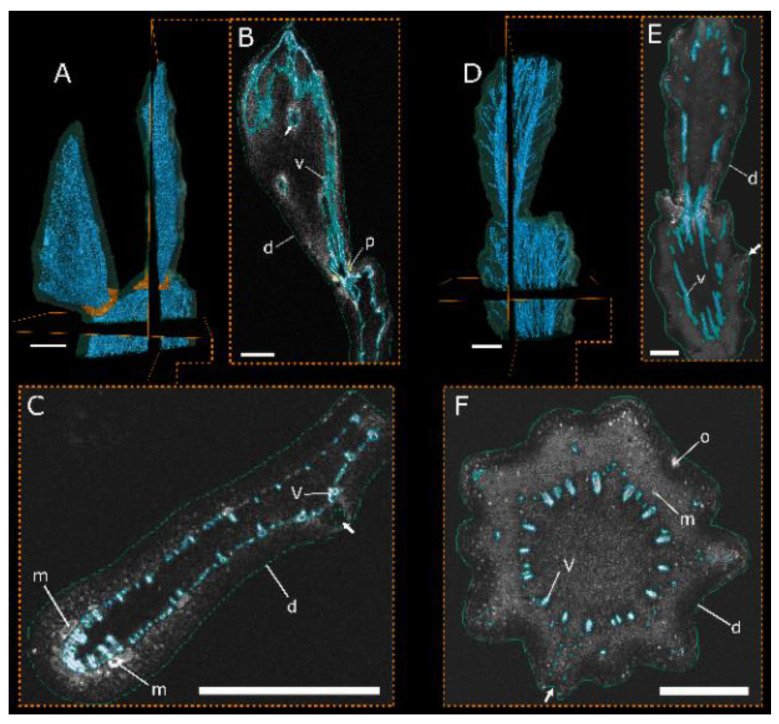
MRI overview scans with exemplary segmentation of the respective slices. Tissue segmentation based on the grey-value signal of the MRI overview scans. Pseudo colour visualisation of the tissues of *Opuntia ficus-indica* (**A**–**C**) and *Cylindropuntia bigelovii* (**D**–**F**) including two sectional planes each ((**B**,**E**): tangential; (**C**,**F**): transverse) for exemplary visualisation of the raw MRI data including segmented areas. d: dermal tissue (green); m: mucilage cell or channel (not segmented); o: oxalate crystal (not segmented); p: peridermal tissue (orange; only in (**A**,**B**)); v: vascular structures (blue). White arrows exemplarily show the locations of the areoles. All scale bars equal 1 cm.

MRI scans were recorded and segmented to obtain a 3D representation of the tissues involved in the branches and their junctions. All scans showed good grey-value contrast between the vascular tissues and the parenchyma. In contrast, the dermal tissue exhibited low contrast to the parenchyma, if any, but could be segmented by a slight contrast difference from the surrounding air (Figure 2). This was not possible for the surface coil scan of *C. bigelovii* because of the low signal of the parenchyma. For this scan, no segmentation of the dermal tissue was performed. The grey levels resulting from the mucilage cells and channels in *O. ficus-indica* and from the mucilage cells and the oxalate crystals in *C. bigelovii* were similar to those of the respective vascular tissues. Therefore, the course of the tissues had to be traced over several slices and was only segmented as vascular tissue in the case of a continuous trajectory (Figure 2C,F).

**Figure 3:**
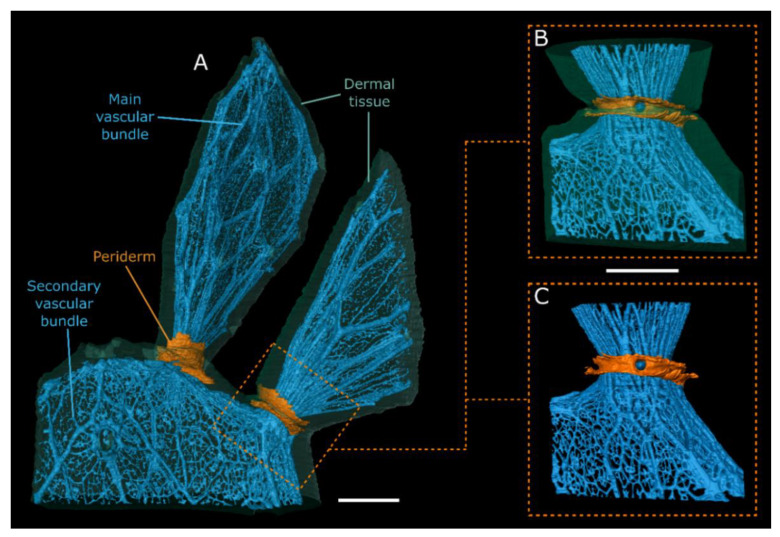
MRI scans of *Opuntia ficus-indica*. 3D representation of the dermal tissue ((**A**,**B**); transparent green), the vascular system ((**A**–**C**); blue) and the peridermal tissue ((**A**–**C**); orange) of a junction site and parts of the connected lateral and sub-lateral branches, based on the segmentation of MRI scans. (**A**) Visualisation (lateral view) of an overview scan (sample 1). Please note that the right and lower branch had to be trimmed because of space limitations of the MRI scanner. Visualisation (lateral view) of the surface coil scan (sample 2) with (**B**) and without (**C**) representation of dermal tissue. All scale bars equal 1 cm.

**Figure 4:**
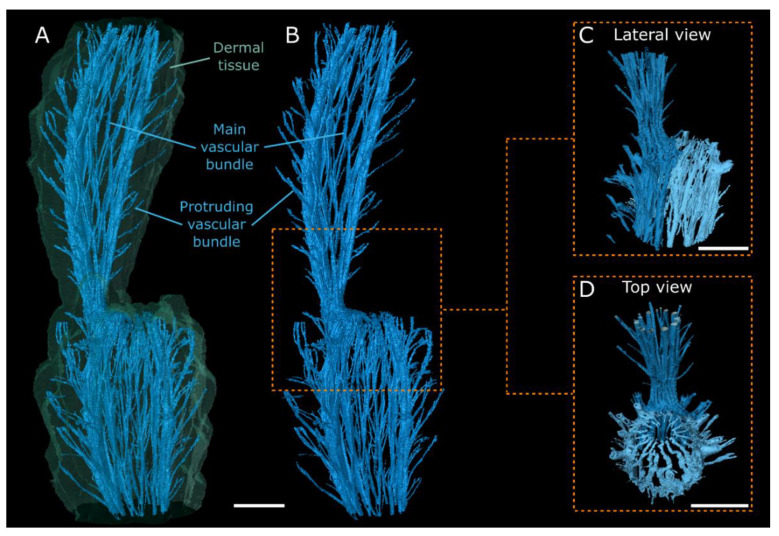
MRI scans of *Cylindropuntia bigelovii*. 3D representation of the dermal tissue (**A**); transparent green) and the vascular system ((**A**–**D**); coloured blue) of the junctions and parts of the connected lateral and sub-lateral branches, based on the segmentation of MRI scans. (**A**,**B**) Visualisation (lateral view) of an overview scan (sample 1). (**C**,**D**) Visualisation of a surface coil scan (sample 2) with a distinction between vascular structures that supply the lateral branch (dark blue) and those that run in a dormant bud (light blue). All scale bars equal 1 cm.

The external outline of the scans provides a good representation of the branch shape visible to the eye: flat branches with slight areolar bulges for *O. ficus-indica* (Figure 3A) and almost cylindrical branches with a large number of undulating areoles for *C. bigelovii* (Figure 4A). Internally, both species exhibit a net-like structure of the vascular bundles. A distinction can be made between the main bundles, which are oriented along the longitudinal axis of the branch, and secondary bundles, which have smaller diameters and run as connections between the main bundles. In addition, both species have bundles that protrude from the net and extend to the areoles. The vascular bundles converge in the junction areas and form an almost closed circular structure with only primary bundles present. Acropetally, they fan out and again form a net-like structure. Whereas the main bundles in *C. bigelovii* (Figure 4A,B) run almost parallel to the branch axis, they form roughly diamond-shaped patterns in *O. ficus-indica* (Figure 3A). In both samples of *C. bigelovii*, about half of the vascular tissue of the sub-lateral branch ran through the junction into the lateral branch (dark blue coloured), while the other half ran in a directly adjacent, dormant bud (light blue coloured; Figure 4). This was not apparent for *O. ficus-indica* for which either two adjacent junction zones were analysed, through which all bundles ran (overview scan, Figure 3A), or a single junction, through which all bundles of the sample ran (surface coil scan) (Figure 3B,C).

In addition to the vascular bundles and the dermal tissue, all three analysed junctions of *O. ficus-indica* exhibited an additional tissue, characterised by the greatest MRI signal (Figure 2A,B): A ring-shaped collar is wrapped directly around the junction of the two branches and, according to macroscopic and microscopic analyses, consists of peridermal tissue. This originates at the basal end of the junction near the vascular bundles and spreads from there laterally and distally, such that, within the junction, it covers major parts of the dermal tissue (Figure 3). In one junction (left in the overview scan; Figure 3A), parts of the periderm also covered lower parts of the lateral branch, whereas in the other two junctions, only the connecting region was covered (right in the overview scan and surface coil scan; Figure 3A–C). No such peridermal tissue with an increased MRI signal was found in the scans of *C. bigelovii*. Animated videos of the segmented overview and surface coil scans for both species are presented in Supplementary Materials Videos S3–S6.

### 3.4. Junction Histology

**Figure 5:**
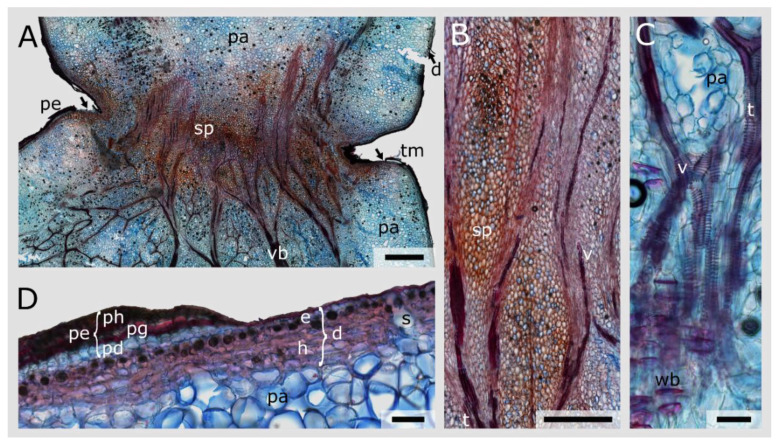
Stained longitudinal microscopic thin sections of a lateral junction of *Opuntia ficus-indica*. (**A**) Overview image showing the vascular bundles (vb), the transition of the parenchyma (pa) to small-volume parenchyma cells (sp) and the peridermal coverage (pe) of the dermal tissue (d). The arrows indicate locations where the periderm detached during preparation of the sections. Trichomes (tm) are visible in the notch of the junction. (**B**) Detailed image of the junction area with small-volume parenchyma (sp) and vascular bundles consisting of vessel elements (v) and tracheids (t). (**C**) Detailed image of tracheids (t), vessels with mostly annular secondary thickening of the cell wall (v) and the less frequent wide-band tracheids (wb) embedded in the large-lumened parenchymatous tissue (pa) and located apical to the junction. (**D**) Detailed image of the dermal tissue (d), consisting of a single-layered epidermis (e), which is covered by a cuticle, and a multi-layered hypodermis (h), which is partly covered by peridermal layers (pe), consisting of densely packed phellem cells (ph), a one-layered meristematic phellogen (pg) and thin-walled phelloderm cells (pd). Stomata (s) and crystalline inclusions (black, circular structures beneath the epidermal layers) are visible. Scale bars: (**A**) 2 mm, (**B**–**D**) 1000 µm.

**Figure 6:**
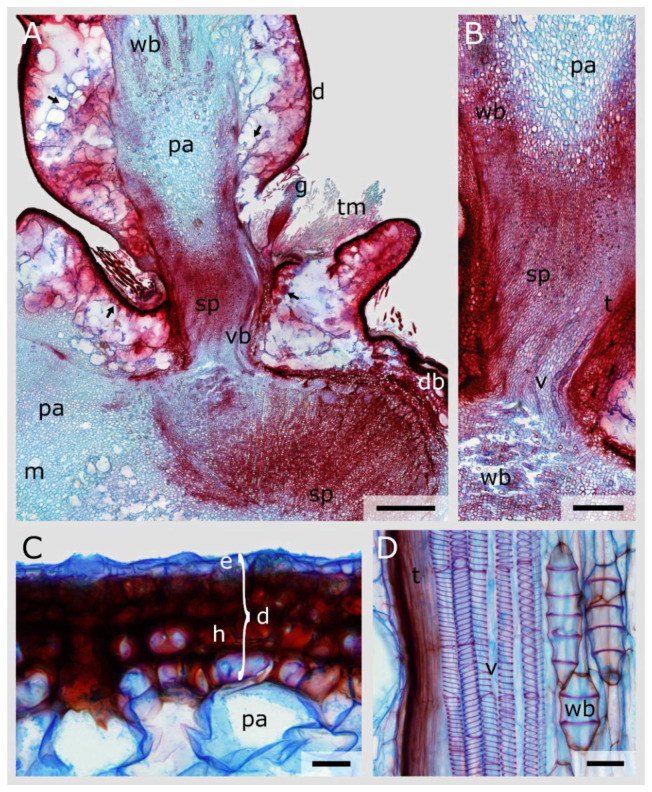
Stained longitudinal microscopic thin sections of a lateral junction of *Cylindropuntia bigelovii*. (**A**) Overview image showing the vascular bundles (vb) partly running through the junction or running into a dormant bud (db), the transition of the parenchyma cells (pa and sp), the dermal tissue (d) and the mucilage cells (m). The arrows indicate locations where the dermal tissue detached from the cortical parenchyma during preparation of the sections. Trichomes (tm) and a glochid (g) are visible in the wrinkle of the junction. (**B**) Detailed image of the junction and the transition between the pith parenchyma (pa) and the small-volume parenchyma (sp). Tracheids (t) and vessel elements (v) run through the junction, whereas wide-band tracheids (wb) are present laterally and basally. (**C**) Detailed image of the dermal tissue (d), consisting of a single layered epidermis (e) covered by a thin cuticle and of a multi-layered hypodermis (h). (**D**) Detailed image of vessel elements with spiral secondary thickening of the cell wall (v), tracheids (t) and wide-band tracheids (wb) embedded in parenchymatous tissue. Scale bars: (**A**) 2 mm, (**B**) 1 mm, (**C**,**D**) 50 µm.

Stained microscopic thin sections were analysed to gain cellular insights into the lateral branch junctions of both species and the periderm formation in *O. ficus-indica*. Both tangential overview images illustrate the geometric constriction between the two branches and the associated reduction of the cross-sectional area (Figure 5A and Figure 6A). A gradual change in parenchymatous tissue becomes apparent across the junctions of the two species. Basally and laterally from the junction, the parenchyma becomes considerably more large-lumened with thin cell walls. Within the junction, the volume of the single cells decreases and inclusions in the cell walls become visible (Figure 5A,B and Figure 6A,B).

The overview section of *O. ficus-indica* reveals that the vascular bundles deviate from their net-like structure to run straight through the junction (Figure 5A). Within the junction, the vascular elements consist mainly of vessels. Basal and apical to the junction, vessel elements and fibres are predominant, but in addition, some wide-band tracheids are embedded in the parenchyma (Figure 5A–C). Trichomes are found growing out of the junction notch (Figure 5A), most of them having fallen off during the preparation of the sections. The dermal tissue of the lateral branches of *O. ficus-indica* consists mostly of a single-cell-layered epidermis, covered with a cuticle, and a hypodermis of three to four layers in thickness. Regularly occurring stomata and crystalline inclusions in the uppermost layer of the hypodermis are visible. Around the junction, a peridermal overlay covers the dermal tissue. It consists of several layers of densely packed phellem, one layer of meristematic phellogen and two to three layers of less densely packed phelloderm (Figure 5D). The preparation of the sections almost always resulted in the tearing off of small pieces of periderm; hence, in some parts (as a preparation artefact), the peridermal coverage appeared not to be continuous across the junction (Figure 5A).

The longitudinal overview section of *C. bigelovii* reveals that the vascular bundles of the sub-lateral branch split into two, with one part passing through the junction and the other running into a dormant bud (Figure 6A). Whereas many wide-band tracheids can additionally be found outside the junction, they are sparse within the junction in which vessels and tracheids predominate (Figure 6A–C).

The dermal tissue of *C. bigelovii* consists of a single-layered epidermis and a four- to five-layered, densely packed hypodermis (Figure 6C). Peridermal coverage is not evident in any of the samples examined. No transition zone is present between the densely packed hypodermis and the wide-lumened, sponge-like parenchyma. Most of the prepared overview thin sections ruptured at this location and, hence, an analysis of the cortex region based on the images was not possible (arrows in Figure 6A). Many trichomes and a glochid were found in the notch of the junction (note its vast constriction of the geometry near the attachment site, which allows for easy shedding) (Figure 6A).

### 3.5. Periderm Formation in Opuntia Ficus-Indica

**Figure 7:**
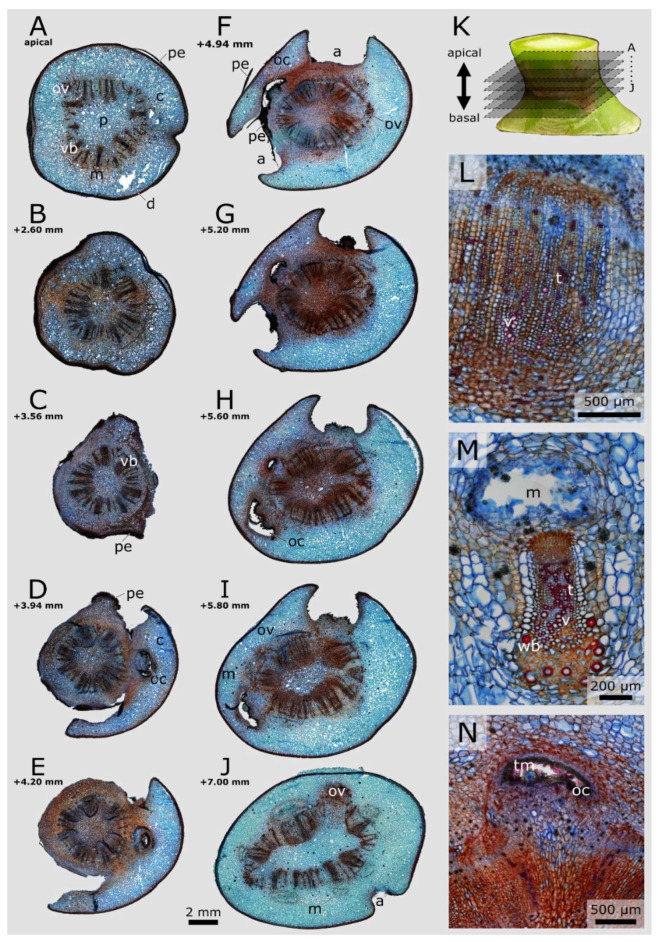
Stained transverse microscopic serial sections of a lateral junction of *Opuntia ficus-indica*. (**A**–**J**) Sections sorted from apical (**A**) to basal (**J**) in the junction, as schematically shown in (**K**). The scale bar applies to subfigures (**A**–**J**). a: areolar protrusion; c: cortex, consisting of parenchyma cells; d: dermal tissue, consisting of an epidermis covered with a cuticle and a hypodermis; m: mucilage channel; oc: outgrowing cavity; ov: outgrowing vascular bundle; p: pith, consisting of parenchyma cells of different sizes and with different cell wall thicknesses; pe: periderm; vb: vascular bundle. (**L**,**M**) Detailed images of the vascular bundles within the junction (**L**) and apical to it (**M**). v: vessel element; t: tracheid; wb: wide-band tracheid. (**N**) Detailed image of an outgrowing cavity (oc) with trichomes (tm).

Serial tangential sections through the junction of *O. ficus-indica* reveal the formation of the areoles and the periderm coverage (Figure 7K). Single vascular bundles separate from their circular arrangement and extend laterally (Figure 7A,F,I,J). At more apical stages, these vascular bundles protrude into outgrowing cavities surrounded by peridermal tissue in which trichomes grow (Figure 7D–I,N). The parenchyma cells surrounding this outgrowing areole also differ from the other cortical parenchyma cells: they are smaller and have inclusions in their cell walls, thus more closely resembling the parenchyma of the junction pith. The xylem and phloem cells, by contrast, are scarce or no longer present. An areole is formed once one of these structures reaches the outer edge of the cross-section (Figure 7F–I). Near the junction, several areoles coalesce, removing an increasing amount of cortical tissue in the apical direction, and form more peridermal tissue (Figure 7C–F). Several pieces of the periderm detached during the preparation of the sections.

The reduction in the cross-sectional area is apparent towards the junction, with little loss of pith area, more densely packed vascular bundles and the loss of mainly cortical tissue (Figure 7B,C). Apical to the junction, the cross-sectional area increases again, and the entire cross-section is covered by periderm. The now near-circular cross-sectional geometry (Figure 7A) becomes oval, and the periderm gradually disappears.

In addition to the periderm development, the cross-sectional images also demonstrate the difference between the leading bundles. Within the junction, they narrow and mostly vessels and tracheids are present. A greater number of wide-band tracheids is found apical to the junction (Figure 7L,M). Mucilage channels are located lateral to the vascular bundles (facing towards the branch periphery) and occur in groups of six in the cross-section just basal to the junction, whereas they disappear completely within the junction and reappear slightly apical to it (Figure 7A,C,J,M).

### 3.6. Tissue Mechanics

Tensile tests were performed to obtain mechanical information of the dermal tissues and vascular bundles found in *O. ficus-indica* and *C. bigelovii*. Because of the limited number of branches per plant that were eligible for the experiments, the experimental groups had to be distributed over two plants each. In total, 36 out of 45 tested variables did not differ significantly between the single plants of one species and could be pooled. However, nine tested variables differed significantly (see, for example, “Deformation at break” of the dermal tissues and the vascular bundles), in which cases the respective values are given for each plant (Table 2 and Table 3).

No significant differences were found between the groups of different orientations (longitudinal and transverse) for the dermal tissues without peridermal layers in *O. ficus-indica*. For the samples of the dermal tissues with peridermal coverage, significant differences between the two orientations were only found for the deformation at break and the Poisson’s ratio (Table 2).

The median thickness of the dermal tissue of *C. bigelovii*, consisting of epidermis and hypodermis, was about 18–20% thinner than their *O. ficus-indica* equivalents. Periderm accumulation in *O. ficus-indica* increased the median thickness by about 17–27% and the tensile strength by a factor of 2.2 to 3.4 to a median of up to 13.7 MPa. The tensile strength values of *C. bigelovii* were significantly higher than those of the *O. ficus-indica* samples without periderm and lay approximately in the range of those with peridermal coverage of *O. ficus-indica*. For the elastic modulus, the values of the dermal tissue of *C. bigelovii* were similar or slightly higher than those of *O. ficus-indica* without periderm. Periderm accumulation increased the elastic modulus of the latter by a factor of about 10 up to a median value of 464.0 MPa. The fracture energy in *O. ficus-indica* samples decreased by a factor of 2.5 to 3.5 as a result of peridermal accumulation, resulting in median values similar to those of *C. bigelovii* (3.3 mJ/mm^2^). The median values of *O. ficus-indica* for deformation at break for groups with periderm were lower (although only partially statistically significant) than those without periderm. The latter, with median values of 15.0 to 20.9%, lay approximately in the same range as those of *C. bigelovii*. No clear trend was found for the values of Poisson’s ratio. The median values of *O. ficus-indica* without peridermal accumulation ranged from 0.52 to 0.65 and those with periderm from 0.21 to 0.64, with a relatively large variation in the data. The values for *C. bigelovii* were lowest with a median of 0.08, being significantly different from all other groups except for the transverse values from one plant of *O. ficus-indica* with peridermal layers (Table 2).

The median diameters of the vascular bundles from the young branches of *O. ficus-indica* were about half the diameter of those of the older branches (0.15 to 0.32 mm), which were both significantly lower than those of *C. bigelovii* with 0.86 and 1.07 mm. The values for the tensile strength, elastic modulus, fracture energy and deformation at break did not differ significantly between the vascular bundles of young and older branches of *O. ficus-indica*. The values were all significantly different from those of *C. bigelovii*: with median values of 56.2 and 51.1 MPa, the tensile strength was about 25 times higher for vascular bundles of *O. ficus-indica*. The median elastic modulus was 165 times and 225 times higher for *O. ficus-indica* with median values of 1233.8 and 1674.0 MPa. For the fracture energy, the median values of *O. ficus-indica* were 16 to 19 times higher than those of *C. bigelovii*, whereas the median values of deformation at break for *C. bigelovii* were about 4 to 12 times higher than those of *O. ficus-indica* (Table 3). The Poisson’s ratio could not be measured for the vascular bundles because of their small initial diameter. Raw data and a detailed statistical comparison for the dermal tissue and vascular bundle analyses can be found in Supplementary Materials Table S11.

In a comparison of the values between the various tissues of the same species, *O. ficus-indica* showed significantly higher values for tensile strength, elastic modulus and fracture energy for the vascular bundles compared with the dermal tissues. No clear trend was observed for the deformation at break values. For *C. bigelovii*, the values of the vascular bundles compared with the dermal tissues were lower for tensile strength, elastic modulus and deformation at break but higher for fracture energy compared with those of the dermal tissues.

## 4. Discussion

The lateral junctions of the Opuntioideae and their range of mechanical behaviour are intriguing research objects for studies on functional morphology. In this comparative work, we analysed the junctions of *O. ficus-indica* and *C. bigelovii* by means of their morphology and anatomy. Additionally, we mechanically characterised the dermal tissues and vascular bundles that mainly influence the mechanical behaviour of the branch–branch junctions. Table 4 provides an overview of the similarities and dissimilarities of various features of the two analysed species. All experiments were conducted on plants that were propagated via offshoots and grown under controlled greenhouse conditions, therefore, the presented results do not reflect the natural diversity of the two species.

### 4.1. Junction Morphology

The tensile, bending and torsional loads that occur in nature, e.g., because of self-weight, external influences such as wind or snowfall [18] or contact with passing mammals [35], can be counteracted by morphological adaptations such as an increase in cross-sections, the axial second moment of area or the torsion constant of the loaded structure [36]. In this context, the external appearance of a plant body can be described and defined by its geometry (e.g., circular, elliptical), its size (e.g., cross-sectional area, axial second moment of area, torsion constant) and its shape (the ratios and gradients between these variables) [37,38].

Both analysed species feature a branch geometry that allows rapid volume increase through water uptake: (1) the oval branches of *O. ficus-indica* can change from an elliptic to a more circular cylinder, reducing the surface to volume ratio and (2) by the flattening of the tubercle protrusions in *C. bigelovii*, an increase in volume can also be enabled while the surface area is maintained [4]. However, the cross-sectional shape of the junctions, indicated by the ratio between *I_major-axis_* and *I_minor-axis_*, is roughly circular and differs only slightly between the two species, so that the resulting stresses are geometrically mainly determined by the size of their cross-sectional area.

The morphometric analyses of both species showed a high decrease in all measured morphometric variables from their branches to their junctions, clearly identifying the junctions as the geometrical weak points under bending, tensile and torsional loading of the systems. Although the cross-sections of the branches differed greatly in geometry between the two species, their cross-sectional area was similar. At 15.4 mm^2^, the median junction area of *C. bigelovii* was within the range measured by Bobich and Nobel [19] and, similar to the area ratio, the axial second moments of area and the torsion constant, was significantly lower than that of *O. ficus-indica*, making *C. bigelovii* junctions more susceptible to mechanical failure (Table 1).

With regard to the axial second moment of area, the orientation with regard to the direction of the acting force plays an additional important role. For instance, the lateral, widely overhanging branches with an elliptical cross-section in *Platyopuntias* can be oriented with their major axis parallel to gravity to increase the axial second moment of area, thereby reducing the resulting bending moments [20]. However, for the slightly elliptical to round junctions of the two species, the orientation with respect to gravity has only a minor effect.

In addition to the geometry, size and shape of the junction, the tissues involved and their localisation and mechanical properties also play a decisive role in determining the stability of the structure. As a non-invasive and non-destructive technique for high-resolution 3D imaging, MRI has proven its suitability for visualising plant tissues over the past few years [39,40,41,42,43] and has previously been used to visualise cactus morphologies [44]. However, the present study is the first work on cacti involving 3D visualisation methods to segment various tissues. This has allowed us to visualise not only the outer branch morphology of both species and the net-like course of their vascular bundles, which converge in the junction to form an almost closed circle without secondary connections, but also the formation of the periderm (as a secondary dermal tissue) in *O. ficus-indica* (Figure 3 and Figure 4). In young stages, the periderm first covers lateral junctions in a collar-like manner before it spreads over the surface of the branches until it completely covers the epidermis (primary dermal tissue) and increases in thickness. Since this accumulation reduces gas exchange and the photosynthetic activity of the green branches, it is vital for the survival of the plant that this process occurs gradually and does not cover all branches in the early stages [1]. In the older stages, however, it can contribute to the continuously increasing need for stability through its layered growth, originating directly at the junction.

### 4.2. Junction Histology

The periderm formation, which in *O. ficus-indica* is a layered tissue on top of the epidermis, represents anatomically the most conspicuous difference between the lateral junctions of the two species. Periderm layers have also been found in self-repair analyses on *O. ficus-indica* and *C. bigelovii* with meristematic phellogen cells differentiating from the cortex parenchyma [28]. The periderm formation sealed the wounds within days and thereby prevented extensive water losses. However, because of its fragile connection with the epidermis, the periderm formation did not help to recover the initial strength and stiffness of the branches. The outgrowth of the lateral branches from the areoles and their subsequent increase in cross-section might lead to internal mechanical stresses and injuries that initiate periderm formation as a secondary coverage. This is also indicated by the cross-sectional images, which show internal cavitation by the expulsion of vascular bundles (Figure 7). One reason that periderm formation was found only in the junctions of *O. ficus-indica* and not in *C. bigelovii* might be the larger junction cross-sectional areas in *O. ficus-indica* leading to junction injuries at young stages.

The cellular composition of the vascular bundles changes in both species within the junctions. No mucilage cells are found within this area, and the parenchymatous tissue is much smaller in the lumen and has thicker cell walls compared with that in the branch (Figure 5 and Figure 6). However, the parenchyma cells in the junctions of *C. bigelovii* are larger compared with those of other species of the Cylindropuntieae [19], leading to a lower stiffness and strength of the tissue [36,45]. All of these changes might have an influence on the mechanical stability and stiffness of the junctions but are likely to play a minor role compared with the other factors presented in this paper.

Abscission, i.e., the shedding of mostly old, diseased or ripe plant organs, is a common phenomenon that can be observed, for example, every autumn when leaves are shed or when ripe fruits fall from trees or shrubs. For this purpose, abscission zones and often also protective layers are formed to protect the developing wounds from infestation and drying out [17]. In addition, the time of abscission is usually determined by hormonal influences and enzyme activities [46]. A protective layer is not found in the Opuntioideae but, as previously mentioned, they possess a good ability to self-heal [28]. A defined abscission zone, which usually consists of a few cell layers of small parenchyma cells, is also not found in the examined species. However, small-lumen parenchymatous cells are present within the entire junction, especially in *C. bigelovii*. Moreover, the described tapering of the cross-sectional area (Table 1 and Figure 6) is another common feature in regions of abscission [47]. A hormonal influence on the shedding of branches has not yet been reported in Opuntioideae. The role of dropping the lateral branches and the associated increase in the reproductive potential of the species can be compared to the dropping of ripe fruits. Thus, in the case of *C. bigelovii*, we can refer to an abscission behaviour with regard to their lateral branches.

### 4.3. Tissue Mechanics

One of the key factors for the mechanical performance of plant structures, in addition to their external geometry and tissue arrangement, are the mechanical characteristics of the individual tissues. Cacti branches, for example, can be well approximated as fibre-reinforced composites with a dermal cover tissue [22,23]. The tensile tests of the dermal tissues of *O. ficus-indica* showed that the orientation (longitudinal vs. transverse) has little to no influence on their mechanical properties (Table 2), i.e., the tissue behaves isotropically in these two dimensions and thus differs from other biological tissues, which behave differently in all three dimensions [36,48,49].

The mechanical values measured for the dermal tissue covered with periderm in *O. ficus-indica* fits well with values for the dermal tissue (‘skin’) in the climbing cactus *Selenicereus setaceus* reported by [50]. Comparing the cacti epidermis (without periderm) with the leaf epidermis of the leaf-succulent species *Delosperma ecklonis* and *Delosperma cooperi* reveals that the tensile strength is by a factor of 3 to 20 higher and the elastic modulus by about a factor of 7 to 30 larger [51]. The higher values of the cacti epidermis can be explained by the densely packed hypodermis. The accumulation of peridermal layers has a major effect on the stiffness of *O. ficus-indica* dermal tissue (with an approximately tenfold increase in elastic modulus) and a modest increase in tensile strength (with a twofold to threefold increase). However, since the periderm tissue fails at lower strain rates, it also lowers the energy needed for fracture (Table 2). The differences between the two investigated species after periderm accumulation were significant for tensile stiffness but much smaller for tensile strength; hence, the dermal tissue composition is probably not the predominant factor for the different failure fracture behaviour of the overall systems. Nevertheless, in much older, tree-like specimens of *O. ficus-indica*, the immense thickening attributable to the accumulation of additional periderm layers might, in addition to the shape change towards circular cross-sections and the growth in thickness mostly occurring as a result of secondary wood formation, have a considerable effect on the stability of the plants.

The differences between the mechanical properties of the vascular bundles of *O. ficus-indica* and *C. bigelovii* were much more pronounced than those between their dermal tissues. The elastic modulus (by a factor of about 200) and strength (by a factor of about 25) (Table 3) are much larger for *O. ficus-indica* than for *C. bigelovii*. Our values for the tensile elastic modulus of the *O. ficus-indica* vascular bundles lie within the wide range of the literature values, namely, from 0.16 GPa [25] through 1.1 GPa [24] to 2.9 GPa [26]. The literature values for strength from the above-mentioned studies are all smaller, at least by a factor of two, than those found in our work. However, since all studies so far have used dried tissue that was not tested on the level of individual fibres but as a network, comparisons between these values have only a limited validity. The elastic modulus and strength of vascular bundles of *O. ficus-indica* are larger and those of *C. bigelovii* are smaller than those found for the central vascular strand of the succulent leaves of two *Delosperma* species [50], results that again highlight the distinct difference of fibre mechanics in the two species of Opuntioideae.

An adaptation or even a dimorphism from wide-band tracheids to mostly vessels and fibres of the vascular bundles [9] of *O. ficus-indica* does not appear to have occurred or is not mechanically measurable at the stages examined. Although the bundles increase significantly in diameter, their mechanical properties remain almost unaffected. The vascular bundles seem to have a decisive influence on the stiffening of the branches of *O. ficus-indica* from early on. For *C. bigelovii*, we were unable to measure the aging process of the vascular bundles because our experimental plants were not of the appropriate age.

In addition, two limitations of our methodology should not be disregarded: (i) the preparation of the vascular bundles exclusively from the attachment site was not possible and so they had to be removed laterally from it; (ii) the cross-section of the bundles was assumed to be circular. By assuming that the oval-shaped bundles were aligned with the longer axis on the microscope slide, we might have (slightly) overestimated their cross-sectional area and thus underestimated the strain values.

## 5. Conclusions

The results of this study show that different concepts for the stabilisation of lateral branch–branch connections have evolved in the Opuntioideae. Because of the outgrowth of the lateral branches from the areoles, the initial geometry and size is dictated by the nature of the areoles. Differences in the young junctions between the two studied species, namely, *O. ficus-indica* and *C. bigelovii*, are therefore mainly found in their size and the composition of the tissues involved. These are evident in the significantly smaller junctions of *C. bigelovii* and the mechanics of the vascular bundles, whose stiffness and tensile strength are 25- to 200-fold smaller compared with those of *O. ficus-indica*. Since the Opuntioideae shed their leaves at a very young stage, photosynthesis is carried out almost exclusively in the branches. An epidermal accumulation of opaque periderm tissue on the branch surface can therefore only contribute to stiffening in older stages, when photosynthesis is of minor importance. However, this covering process starts early in young branches of *O. ficus-indica* around the junction, presumably induced by self-repair processes after injuries during branch outgrowth from the areoles. The aging processes of the branches are accompanied not only by an increase in the size of the junction but also by the growth of the lateral branches and thus an increase in the resulting bending moments. Whereas *O. ficus-indica* progresses to tree-like growth forms through increasingly rounded stems and (main) branches, the growth of junctions and the accumulation of periderm, these adaptations are much less pronounced in *C. bigelovii*, a species that reaches maximum shrub height and in which the abscission of branches enables and is important for vegetative reproduction.

The insights gained from this study can be used as a basis for numerical simulation approaches aiming for a better understanding of the temporally and spatially controlled mechanical behaviour of plant branching. Such approaches will allow researchers to quantify the influence of relevant structural features that determine bonding or debonding properties in complex biological materials systems in general. The combined results of our experimental studies and the findings from future simulations may serve as basis for the development of engineered materials systems with bonding–debonding mechanisms. Plant-inspired bonding can help to extend the service life of products, and plant-inspired debonding can facilitate material separation and recycling at the end of the service life, which would be a valuable contribution to increase sustainability.

## Figures and Tables

**Table 1:** Morphometric comparison of lateral branches and their junctions in *Opuntia ficus-indica* and *Cylindropuntia bigelovii*. The junction area was measured directly at the attachment point after the twisting off of the branch. The cross-section of the branches was measured at its widest point. The area ratio was calculated according to Equation (1). The axial second moments of area (*I_major-axis_* and *I_minor-axis_*), their ratio and the torsion constant *K* (according to Equations (2) and (3)) were analysed along the major and minor axes of the respective cross-sections. The statistical test to check for differences between the two species was chosen depending on whether a normal distribution of the data and data with equal variances were given (^a^: Welch two-sample *t*-test) or not given (^b^: Wilcoxon rank sum test). n.s. (not significant) *p* ≥ 0.05, * 0.05 > *p* ≥ 0.01, ** 0.01 > *p* ≥ 0.001, *** 0.001 > *p*.

	*O. ficus-indica*	*C. bigelovii*	Statistical Difference *p*-Value & Significance Level
Sample Size *N*	12	12	
Morphometric Variable	Median (IQR)	Median (IQR)	
Junction area	25.02	15.42	0.0068
[mm^2^]	(15.10)	(7.22)	** ^b^
Branch area	382.09	395.58	0.4713
[mm^2^]	(138.04)	(103.41)	n.s. ^a^
Area ratio	0.068	0.038	0.0011
[ ]	(0.040)	(0.012)	** ^b^
Junction *I_major-axis_*	43.86	19.56	0.0056
[mm^4^]	(37.42)	(18.62)	** ^b^
Junction *I_minor-axis_*	56.96	26.55	0.0173
[mm^4^]	(45.66)	(29.02)	* ^b^
Junction *I_major-axis_/I_minor-axis_*	0.851	0.717	0.0678
[ ]	(0.203)	(0.087)	n.s. ^a^
Branch *I_major-axis_*	2045.41	12,600.93	>0.0001
[mm^4^]	(1207.33)	(6356.29)	*** ^b^
Branch *I_minor-axis_*	98,875.21	13,321.64	>0.0001
[mm^4^]	(35,309.61)	(7028.65)	*** ^b^
Branch *I_major-axis_/I_minor-axis_*	0.021	0.935	>0.0001
[ ]	(0.017)	(0.029)	*** ^a^
Ratio *I_major-axis_*: junction/branch	0.02606	0.00133	>0.0001
[ ]	(0.04370)	(0.00068)	*** ^b^
Ratio *I_minor-axis_*: junction/branch	0.00081	0.00192	0.0014
[ ]	(0.00033)	(0.00063)	** ^a^
Junction torsion constant *K*	110.78	43.21	0.01
[mm^4^]	(81.76)	(30.94)	* ^b^
Branch torsion constant *K*	4776.45	24,909.54	>0.0001
[mm^4^]	(2854.11)	(13,002.07)	*** ^b^
Ratio torsion constant:	0.0222	0.00017	>0.0001
junction/branch [ ]	(0.05398)	(0.00010)	*** ^b^

**Table 2:** Mechanical properties of dermal tissues of *Opuntia ficus-indica* and *Cylindropuntia bigelovii* obtained by tensile tests. Dermal tissue consists of the outer epidermis and the multilayered hypodermis. The samples of *O. ficus-indica* were taken in both the transversal and longitudinal directions. Limitations were experienced in the sampling of *C. bigelovii* because of the large number of tubercles, although they can be considered as approximately “longitudinal”. Median and IQR values are given for all variables for better comparability, regardless of whether a normal distribution of the data was found. Statistical differences between individual plants led to the division of data in the same group. Capital letters for each row indicate the groups without statistically significant differences in the post-hoc tests (paired Wilcoxon test) for the respective variable. Detailed group-wise statistical comparisons can be found in Supplementary Materials Table S9.

Tissue	*O. ficus-indica*							*C. bigelovii*	
	Dermal Tissue			Dermal Tissue Covered with Periderm				Dermal Tissue	
Sample Orientation	Longitudinal	Transverse		Longitudinal		Transverse		“Longitudinal”	
Sample Size *N*	12	12		12		12		12	
		6	6	6	6	6	6	6	6
Mechanical Variable	Median (IQR)	Median (IQR)		Median (IQR)		Median (IQR)		Median (IQR)	
Thickness [mm]	0.22 ^A^	0.20 ^A^		0.26 ^B^		0.27 ^B^		0.16 ^C^	
	(0.03)	(0.06)		(0.07)		(0.04)		(0.02)	
Strength [MPa]	4.08 ^A^	3.96 ^A^		12.21 ^B^	8.83 ^BC^	13.36 ^B^		6.23 ^C^	11.21 ^B^
	(0.86)	(0.72)		(1.54)	(2.96)	(5.72)		(1.29)	(4.39)
Elastic modulus [MPa]	44.09 ^AB^	40.76 ^A^		463.95 ^C^		414.45 ^C^		39.51 ^AB^	68.68 ^B^
	(13.73)	(15.59)		(179.48)		(256.97)		(17.95)	(57.09)
Fracture energy [mJ/mm^2^]	11.94 ^A^	11.31 ^A^		4.82 ^B^		3.20 ^B^		3.30 ^B^	
	(2.69)	(3.68)		(4.08)		(2.06)		(1.84)	
Deformation at break [%]	17.24 ^A^	20.90 ^A^	15.00 ^A^	4.54 ^B^		10.16 ^AC^	4.49 ^BC^	19.51 ^A^	
	(3.41)	(2.72)	(2.23)	(2.32)		(1.37)	(0.81)	(8.78)	
Poisson’s ratio [ ]	0.65 ^A^	0.52 ^AB^		0.34 ^AC^		0.21 ^BCDE^	0.64 ^AD^	0.08 ^E^	
	(0.21)	(0.33)		(0.22)		(0.23)	(0.18)	(0.09)	

**Table 3:** Mechanical properties of the vascular bundles of *Opuntia ficus-indica* and *Cylindropuntia bigelovii* obtained by tensile tests. Median and IQR values are given for all variables for better comparability, regardless of whether a normal distribution of the data was found. Statistical differences between individual plants led to the division of data in the same group. Capital letters for each row indicate the groups without statistically significant differences in the post-hoc tests (paired Wilcoxon test) for the respective variable. Detailed group-wise statistical comparisons can be found in Supplementary Materials Table S10.

Tissue	*O. ficus-indica*			*C. bigelovii*	
	Vascular Bundle	Vascular Bundle		Vascular Bundle	
	(Young Branch)	(Older Branch)			
Sample Size *N*	12	12		12	
		6	6	6	6
Mechanical Variable	Median (IQR)	Median (IQR)		Median (IQR)	
Diameter [mm]	0.153 ^A^	0.321 ^B^		0.856 ^C^	1.068 ^D^
	(0.105)	(0.101)		(0.167)	(0.399)
Strength [MPa]	56.24 ^A^	51.11 ^A^		2.09 ^B^	
	(56.52)	(35.75)		(1.25)	
Elastic modulus [MPa]	1233.83 ^A^	1673.97 ^A^		7.44 ^B^	
	(1061.79)	(384.52)		(2.89)	
Fracture energy [mJ/mm^2^]	36.20 ^A^	41.90 ^A^		2.25 ^B^	
	(48.82)	(63.03)		(1.56)	
Deformation at break [%]	10.86 ^A^	12.21 ^A^	5.33 ^A^	65.39 ^B^	44.11 ^B^
	(7.36)	(3.36)	(5.70)	(24.54)	(10.99)

**Table 4:** Overview of the similarities and dissimilarities comparing various features of *Opuntia ficus-indica* and *Cylindropuntia bigelovii*.

Feature	*O. ficus-indica*	*C. bigelovii*
**Life-Form**		
Growth-form	stem succulent	
	tree-like	shrubby
Main propagation	sexual via fruits/seeds	vegetative via shed offshoots
Branch–branch junction stability	stable	fragile (abscission)
**Similarities**		
Morphometry	distinct cross-sectional taper towards the junctions	
Morphology	net-like arrangement of vascular bundles that close together circularly within the junctions	
Anatomy	changes in tissue characteristics from the branches to the junctions: lumen of parenchyma changes from large to small and the amount of wide-band tracheids changes to a smaller number	
Mechanical properties	comparable stiffness of the dermal tissues (without periderm coverage)	
**Dissimilarities**		
Morphometry	*C. bigelovii:* significantly lower absolute and relative junction cross-sectional areas	
Morphology	*O. ficus-indica:* periderm formation around lateral junctions	
Anatomy	*O. ficus-indica:* periderm formed as wound tissue by outgrowing areoles	
Mechanical properties	*O. ficus-indica:* periderm stiffens dermal tissue by a factor of about 10	
	*O. ficus-indica:* strength and stiffness of vascular fibres are higher by a factor of about 25 and 200, respectively	

## Data Availability

All data needed to evaluate the conclusions in the paper are present in the paper and/or the electronic supplementary material. Additional data related to this paper may be requested from the authors.

## Supplementary Materials

The following are available online at https://www.mdpi.com/article/10.3390/plants10112313/s1, Document S1: DNA Sequencing—Material and Methods, Primer and Results, Document S2: Detailed anatomical methods, Video S3: Animated overview MRI video of *O. ficus-indica*, Video S4: Animated surface coil MRI video of *O. ficus-indica*, Video S5: Animated overview MRI video of *C. bigelovii*, Video S6: Animated surface coil MRI video of *C. bigelovii*, Table S7: MRI scanning settings, Table S8: Raw data and statistics of morphometric quantification, Table S9: Group-wise statistics of dermal tissues, Table S10: Group-wise statistics of vascular bundles, Table S11: Raw data and statistics of micro-tensile testing.
