# Supplementary material for: Morphology and Anatomy of Branch–Branch Junctions in Opuntia ficus-indica and Cylindropuntia bigelovii: A Comparative Study Supported by Mechanical Tissue Quantification
Source: Plants (Basel). 2021 Oct 27;10(11):2313. doi: 10.3390/plants10112313 (PMC8618873; doi:10.3390/plants10112313)
Supplement: Supplementary file 1 [file plants-10-02313-s001.zip › Supplement 1_DNA Sequencing- Primer & Results.pdf]

Supplement 1: Molecular genetic determination

Opuntia Junctions

Mylo *et al*., 2021 - Plants

Table 1.1: ITS-primers used in PCR for molecular determination of plant sample

Name of primer	Sequence (5'-3')
p5 (forward, 18S, plant-specific)	CCTTATCAYTTAGAGGAAGGAG
u4 (reverse, 26S, universal)	RGTTTCTTTTCCTCCGCTTA

Results

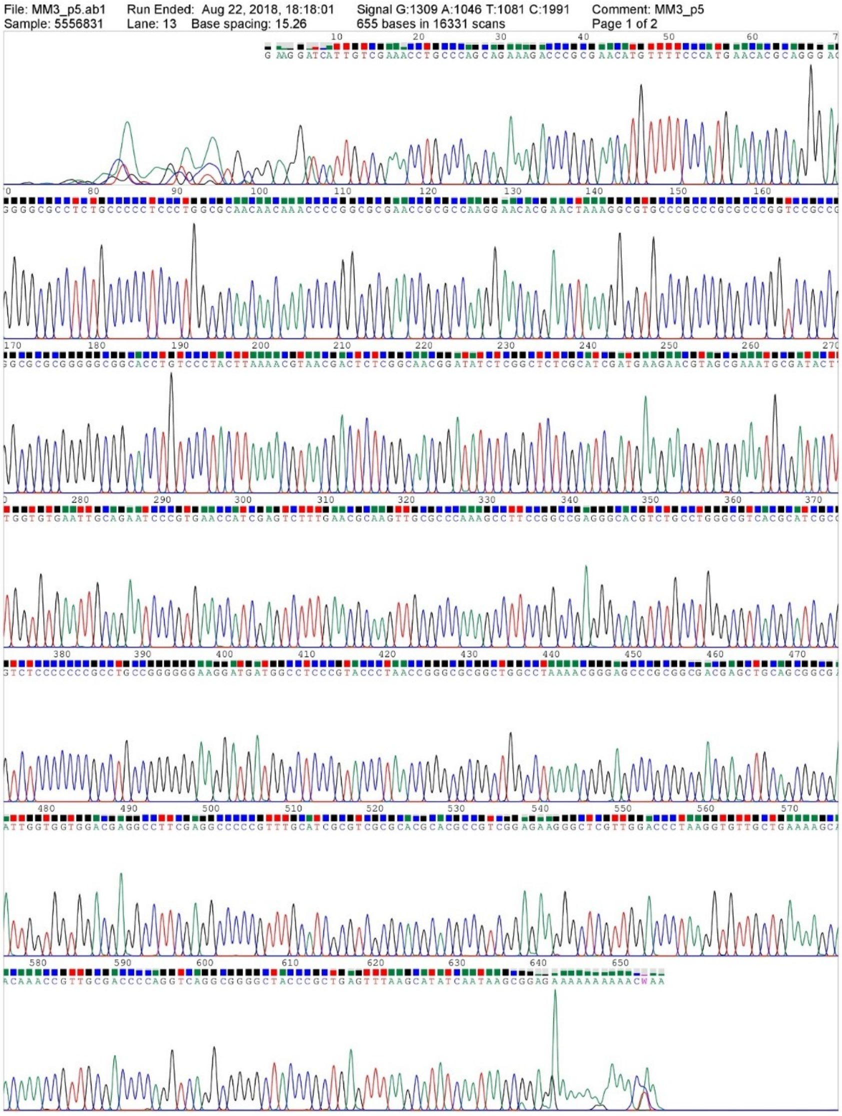

Figure 1.2: Chromatogram of sequenced PCR-product, image created with Chromas 2.6.6 (Technelysium).

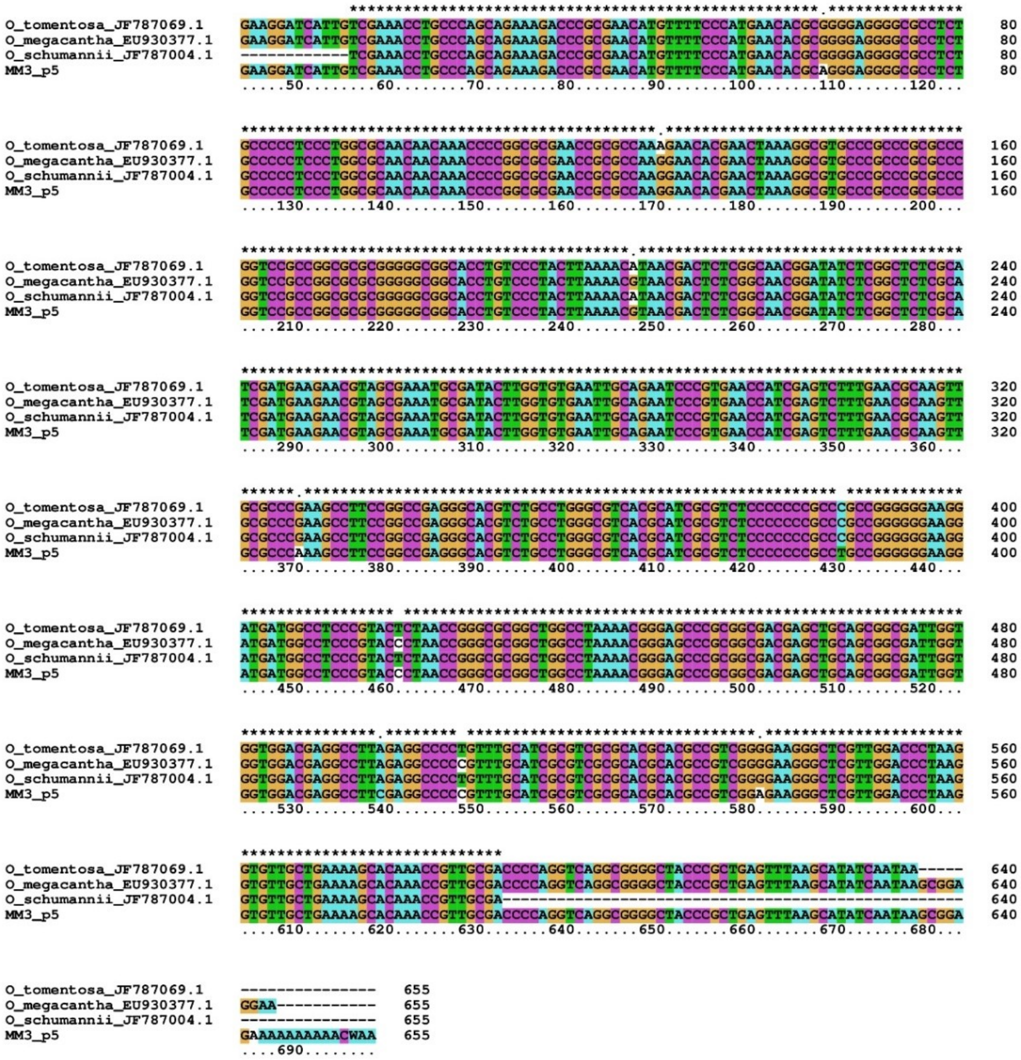

Figure 1.3: Alignment of the sample's ITS-region (MM3_p5) to those of closely related species. Database accession numbers are given after each species name. Picture created with ClustalX (www.clustal.org).
