# Supplementary material for: Morphology and Anatomy of Branch–Branch Junctions in Opuntia ficus-indica and Cylindropuntia bigelovii: A Comparative Study Supported by Mechanical Tissue Quantification
Source: Plants (Basel). 2021 Oct 27;10(11):2313. doi: 10.3390/plants10112313 (PMC8618873; doi:10.3390/plants10112313)
Supplement: Supplementary file 1 [file plants-10-02313-s001.zip › Supplement 2_Detailed anatomical methods.pdf]

# Supplement 3: Detailed anatomical methods

***Opuntia* Junctions**

Mylo *et al.*, 2021 - Plants

Sections of *O. ficus-indica* were obtained from the junction zone between sublateral to lateral branches. The sample from the MRI surface coil scans was used for the analyses of the longitudinal sections directly after the scans. A second sample was used for serial transverse sections. The lateral branch of both samples showed periderm formation only at the junction but not on the stem surface. The samples were covered with embedding medium (Tissue-Tekfi O.C.T.™ Compound, Sakura Finetek Europe B.V., Alphen aan den Rijn, Netherlands) and frozen for at least two hours in a floor-standing cryostat (MEV, SLEE medical GmbH, Mainz, Germany). Sections with a thickness of 60 µm to 70 µm were cut by using the integrated microtome blade of the cryostat. For the longitudinal samples, sections were analysed that went approximately through the centre of the sample. For the transversal serial cuttings, sections from about 5 mm basal to about 5 mm apical of the junction were analysed. In the more apical or basal parts, every third section was examined and in the area of the junction, every section was analysed, resulting in a total number of 77 transverse sections. Cuttings were transferred to a 50% bleach solution (Eau du Javel, Floreal Haagen GmbH, Wadgassen, Germany) for about 15 min to remove mucilage before being transferred into distilled water. For contrast staining, the cuttings were dipped into a safranin O solution (1 g safranin O in 100 mL distilled water, red, staining of lignified cell walls as well as nuclei) for two to three seconds, rinsed in distilled water, stained in an Astra blue solution (0.5 g Astra blue in 100 mL of 2% aqueous Tartaric acid, staining of polysaccharides of the cell wall such as cellulose and pectins) for twelve to fifteen seconds and again washed in distilled water. To create a high-contrast and comparable staining, this last dyeing and washing step was repeated.

Fresh samples of *C. bigelovii* were dehydrated in ascending concentrations of isopropanol and finally embedded in a mixture of 2 parts hydroxyethyl methacrylate and 1 part polyethylene glycol distearate. The medium was polymerized at 60 °C for 12 hours, using benzoyl peroxide (0.5 %) as a catalyst. After hardening, the samples were cut to a thickness of 3 µm in a rotation microtome (RM 2065, Leica, Wetzlar, Germany) with a diamond blade. The sections were then transferred to gelatin-coated slides and the embedding medium dissolved out again in pure isopropanol overnight. The staining process took place automated (in a HMS Series Programmable Slide Stainer, Zeiss, Oberkochen, Germany) as following: 12 h in Safranin (2 %, red, staining of lignified cell walls as well as nuclei) and acriflavine (1 %, yellow, DNA), after rinsing with tap water followed by 30 min of acid yellow (1 %, proteins and nuclei). After rinsing again, methylene blue (1 %, membranes, (protein rhich) plasma, tannin-rich cells) was used as complementary staining for another 5 min. Once rinsed and dried, the sections were covered with DPX Mountant for Histology (Sigma Aldrich, St. Louis, MO, USA) and a cover slip to make them durable.
