# Supplementary material for: Morphology and Anatomy of Branch–Branch Junctions in Opuntia ficus-indica and Cylindropuntia bigelovii: A Comparative Study Supported by Mechanical Tissue Quantification
Source: Plants (Basel). 2021 Oct 27;10(11):2313. doi: 10.3390/plants10112313 (PMC8618873; doi:10.3390/plants10112313)
Supplement: Supplementary file 1 [file plants-10-02313-s001.zip › Supplement 7_MRI scanning settings.pdf]

Detailed MRI scanning parameters

Opuntia Junctions

Mylo et al., 21021 - Plants

**Species**	*Opuntia ficus-indica*	*Opuntia ficus-indica*	*Cylindropuntia bigelovii*	*Cylindropuntia bigelovii*
**Scanner**	Bruker Biospec 94/20	Bruker Biospec 94/20	Bruker Biospec 94/20	Bruker Biospec 94/20
**Coil**	Tx/Rx quadrature volume coil	Tx/Rx quadrature volume coil	Tx/Rx quadrature volume coil	Tx/Rx quadrature volume coil
**Receive only surface coil**	no	yes	no	yes
**Sequence**	3D Flash	3D Flash	3D Flash	3D Flash
**Repetition time [ms]**	30	24	30	24
**Echo time [ms]**	3.9	3.5	3.9	3.8
**Alpha [°]**	30	20	30	35
**Averages**	2	8	4	8
**Duration**	2h34min	13h33min	12h18min	13h33min
**Matrix**	800 x 400 x 320	600 x 420 x 420	920 x 600 x 512	800 x 400 x 320
**Field of View [mm³]**	80 x 60 x 51.2	40 x 28 x 28	90 x 60 x 51.2	48 x 28 x 28
**Resolution [µm³]**	100 x 150 x 160	67 x 67 x 67	100 x 100 x 100	67 x 67 x 67
**AA**	1 1.2 1	1 1.2 1.2	1 1.2 1	1 1.2 1.2
